# Oncologic and visual outcomes after postoperative proton therapy of localized conjunctival melanomas

**DOI:** 10.1186/s13014-019-1426-6

**Published:** 2019-12-27

**Authors:** Juliette Thariat, Julia Salleron, Celia Maschi, Edouard Fevrier, Sandra Lassalle, Lauris Gastaud, Stephanie Baillif, Audrey Claren, Florent Baumard, Joel Herault, Jean Pierre Caujolle

**Affiliations:** 1 Department of Radiation Oncology, Francois Baclesse Cancer ARCHADE Center, Normandie Universite-Unicaen, 3 Av General Harris, 14000 Caen, France; 2 Laboratoire de physique corpusculaire IN2P3/ENSICAEN - UMR6534, 3 Av Genenral Harris, 14000 Caen, France; 3 Department of Biostatistics, Institut de Cancérologie de Lorraine, Université de Lorraine, F-54500 Vandœuvre-lès-Nancy, France; 4 Department of Ophthalmology, Pasteur 2 Teaching Hospital, Nice, France; 5 Department of biopathology, Pasteur 2 Teaching Hospital, Nice, France; 6 Department of Medical Oncology, Antoine-Lacassagne Cancer Center, Nice, France; 7 Department of Radiation Oncology, Antoine-Lacassagne Cancer Center, Nice, France

**Keywords:** Conjunctiva, Melanoma, Treatment, Surgery, Mitomycin, Irradiation / proton therapy, Vision

## Abstract

**Introduction:**

conjunctival melanomas have high local relapse rates. Oncologic and visual outcomes can be improved with proton therapy and no-touch surgery.

**Material and methods:**

a monocentric retrospective study of consecutive patients treated with surgery and proton therapy for conjunctival melanoma was conducted. Proton therapy was performed to a total dose of 45 Grays physical dose delivered in eight fractions over two weeks.

**Results:**

Ninety-two patients were included. The mean age was 63-year-old. 65.2% of patients had primary acquired melanosis. The mean tumor thickness and diameter was 2.5 mm and 7.0 mm respectively. The clinical stage was T1 in 71.6% of cases, with a quadrangular involvement of more than 90° in 69% of cases. Conjunctival melanomas were of epithelioid cell-type in 40% of cases. Mean follow-up was 4.7 years. Five-year local failure rate was 33.2%. Of 25 local recurrences, 14 were marginal/out-of-field, 4 in-field, others were undetermined. First surgery at expert center resulted in 24.3% of local failure at 5 years versus 38.7% if performed elsewhere (*p* = 0.41). Salvage exenteration was performed in 13 patients. Tumor stage and quadrangular involvement were significant factors for local failure. Five-year progression-free survival and cause-specific death rates were 61.5 and 3.6%. Stage and epithelioid type were associated with poorer progression-free survival. Trophic toxicity occurred in 22.9% of patients and was treated locally, with grafts in 7 patients. Glaucoma and cataract occurred in 13 and 22 patients respectively. Prognostic factors for visual deterioration were age, tumor extent (multifocality, quadrangular involvement > 180°) and cryotherapy.

**Conclusions:**

5-year local failure rate after postoperative proton therapy for conjunctival melanoma was of 33.2%. Radiation-induced complications were overall manageable.

## Introduction

Conjunctival melanomas are rare but deadly tumors of the ocular surface. Substantial increase in incidence of conjunctival melanoma has been reported in the last decades with now up to 0.8 cases per million inhabitants [1, 2] in Caucasians. There is however some racial and ethnic variability [3]. Their changing incidence patterns coincide with those seen in cutaneous melanoma, suggesting a possible link to a sunlight-related etiology. Local recurrences are reported in up to 50% of patients at 10 years and metastatic disease may occur via both lymphatic channels to regional pre-auricular lymph nodes and parotid nodes [4] and hematogenously to distant metastatic sites [2, 5]. An eye-preserving strategy is advocated if intraocular and orbital structures are not involved [6]. In early local disease, the mainstay of treatment is the standard “no touch” surgical technique, which consists of removing the tumor with clear margins without touching the tumor [7, 8]. It may be associated with absolute alcohol corneal epitheliectomy in case of corneal involvement and cryotherapy of the cut conjunctival edge [9, 10]. Local relapses may be further minimized by adjuvant therapies [11], such as topical antimitotic agents (mitomycin C, 5-fluorouracil or interferon alpha-2b). However, topical drugs have limited penetration depth and limited efficacy in deeply invasive melanomas. Moreover, distant metastases and mortality are highly correlated with local conjunctival recurrence [5], suggesting that more aggressive local treatment should be necessary [10]. Adjuvant conjunctival radiotherapy can be performed using proton therapy, brachytherapy or electron beam radiation therapy [12–15].

We evaluated the patterns of failure, progression-free survival and prognostic factors, toxicity and visual outcomes in patients with conjunctival melanomas referred to our tertiary-care institution and all treated with surgery and proton therapy.

## Material and methods

### Study population

This single-tertiary care center institutional Review Board approved retrospective case series adhered to the tenets of the Declaration of Helsinki. It included medical records of consecutive patients with histology-proven conjunctival melanomas treated with surgery and proton therapy at the Department of Ophthalmology from 1992 to 2018.

### Data collection

Clinical data included age, gender, ethnicity, laterality, symptoms and previous history of melanocytic tumor, primary acquired melanosis (PAM) and nevus. Tumor characteristics included: conjunctival location (bulbar conjunctiva with or without involvement of the limbus, tarsal conjunctiva or caruncle), extent into adjacent tissues (corneal and/or scleral involvement, anterior chamber and/or orbital extension), conjunctival location in quadrants (nasal, temporal, superior or inferior), tumor size on physical examination in degrees (less than 90°, between 90° and 180° or more than 180° of corresponding limbal circumference) and maximum thickness measured by optical coherence tomography or ultrasound biomicroscopy. Tumors were staged according to the American Joint Committee on Cancer (AJCC) Cancer Staging Manual, 8th Edition [16–18]. Histological analysis was made by a senior onco-ophthalmology pathologist. Marker silk sutures on excised tissues indicated excised tissue orientation. Several serial cutting lengths of paraffin-embedded tissue were analyzed perpendicular to the major tissue axis in order to analyze the epithelium and chorion on the whole excised tissue. Previous history of melanocytic tumor was recorded.

Therapeutic modalities, quality of resection, conjunctival reconstruction (conjunctival, amniotic membrane or mucous membrane grafts) and complications were recorded. An onco-ophthalmologist performed the no-touch technique, associated with corneal epitheliectomy in case of clinical corneal involvement [19]. Double freeze-thaw cryotherapy (perioperatively) of the excision margins and mitomycin on the ocular surface were performed at the discretion of the surgeon. Cryotherapy was progressively abandoned due to atrophy of the cornea and sclera [20]. Mitomycin (when used) was started one month after postoperative irradiation to avoid the cumulative acute toxic irritative effects of both irradiation and mitomycin. It was delivered at a concentration of 0.04% for two 15-day courses with one week-interruptions to recover from mitomycin-induced toxicities.

Proton therapy was performed to a total dose of 45 Grays (Gy) physical dose in eight fractions delivered over two weeks (from Tuesday of the first week to Friday of the second week), starting 1 to 3 weeks if possible per standard practice but it could be more in case of postoperative complications and time to pathology reports It included all areas of invasive melanoma. PAM was not included in the radiation fields in the absence of invasive melanoma. In case of large lesions with macroscopic and microscopic components, a two-step treatment was used with a large field including the full quadrants from limbus to conjunctival folds to 31.2 Gy and a reduced boost to the macroscopic tumor for 13.8 additional Gy [21]. Until 2016, all conjunctival lesions were treated with four tantalum fiducials placed per-operatively at the borders of the operative bed, with one fiducial targeting the conjunctival fold. After 2016, strictly limbal lesions were treated without fiducial. These fiducials were used to accurately define tumor extents and corresponding radiation fields. When needed, an individually shaped compensator was brought into the beam to modify the range of the protons so that the eye was irradiated only at a depth of 2 mm. A brass collimator shaped the beam laterally to have 2.5 mm lateral margins around the involved conjunctiva. In soft tissues, a similar margin was used. However, the distal margin was conformed using a compensator to limit the dose to intraocular structures. The eyelids were spared from the radiations unless involved. Fixation was optimized for preservation of ocular structures. The Eyeplan treatment planning system software was used.

A local recurrence was defined as the appearance of a new clinical lesion which was not present on immediately postoperative slit lamp photography. Matching between treatment plan and site of relapse was performed whenever initial and failure photographs were available and could be co-registered with proton therapy plan. Local relapse was then defined as in-field if within the 90% isodose line or out-of-field if out of the collimators or marginal in between in-field and out-of-field. Salvage treatments of local relapses were reported. Visual acuity was recorded in Snellen scale and converted into logMAR for comparison of visual outcomes with baseline visual acuity. Toxicities were reported according to Common Toxicity Classification of Adverse Events (CTCAE) v4 classification.

### Statistical analysis

Quantitative parameters were described by median, mean and standard deviation, qualitative parameters by frequency and percentage. Incidence of local relapse was described with the Fine and Gray model, to take into account competing risks such as emergence of metastases or death whatever the cause. The Kaplan–Meier method was performed to describe progression free survival (PFS) defined as the time lapse between the date of diagnosis and the date of relapse or death, whatever the cause. The prognostic value of each factor on local relapse was studied using the bivariate Fine-Gray model, and the results were expressed with the hazard ratio (HR) and its 95% confidence intervals. The parameters with a *p*-value less than 0.1 in bivariate analysis were introduced in a multivariate Fine-Gray model, with backward selection. The same process was performed to investigate prognostic factors of PFS by using the Cox proportional-hazards model.

The difference of visual acuity between baseline and follow-up was computed for each patient. Prognostic factors of visual acuity deterioration were investigated with linear regression by adjusting on the baseline value. The parameters with a *p*-value less than 0.1 in bivariate analyses were introduced in a multivariate linear regression with backward selection. The linearity assumption was checked by inspecting the Residuals vs Fitted plot. The QQ plot of residuals was used to visually check the normality assumption. The Cook’s distance was computed to determine the influence of a value.

All statistical analyses were performed using SAS software (SAS Institute Inc., Cary, NC 25513). *P*-values < 0.05 were considered statistically significant.

## Results

After exclusion of seven patients who had surgery only or proton therapy only for comorbidities and age (*N* = 4) or unresectable disease (*N* = 3), the remaining 92 patients all underwent surgery and proton therapy. Patients and tumor characteristics are presented in Table 1. Patients had stage T2 to T3 conjunctival melanoma in 28.4% of cases and over two thirds of them had more than one quadrant involved. A majority of patients (65.2%) had conjunctival melanoma with PAM. Forty-two patients (47.2%) had their first surgery at our hospital. Adjuvant treatments other than proton therapy varied over time with cryotherapy performed systematically until 2009 and mitomycin delivered postoperatively after 2010 based on operative findings and histological reports: respectively 44 (47.8%) and 22 (23.9%) patients either received cryotherapy perioperatively or mitomycin postoperatively, the remaining 28.3% receiving surgery and proton therapy only. Among patients receiving mitomycin, 90.9% had PAM.

**Table 1:** patient and tumor characteristics

Patient and tumor characteristics	N (%) or mean for continuous variables
Sex	
Male	51 (55.4%)
Female	41 (44.6%)
Age	63.0 [48.7–72.0]
Precancerous lesion	
de novo naevus	19(20.7%) 13 (14.1%)
PAM	60 (65.2%)
Clinical stage	
T1	63 (71.6%)
T2	13 (14.8%)
T3	12 (13.6%)
Pathological stage	
pT1	63 (72.4%)
pT2	14 (16.1%)
pT3	10 (11.5%)
Unifocal	76 (82.6%)
Tumor epicenter bulbar	77 (84.6%)
caruncle and conjunctival folds + lids	14 (15.4%)
Quadrangular involvement	
< 90 degrees	26 (31.0%)
90 to 180 degrees	50 (59.5%)
> 180 degrees	8 (9.3%)
Epithelioid type	32 (39.5%)
Margins	
R0	38 (41.8%)
R1	44 (48.3%)
R2	9 (9.9%)
Number of mitoses	3 [0–11]
Ulceration	12 (14.8%)
Lymphatic emboli	2 (2.4%)
Vascular emboli	1 (1.2%)
Thickness (mm)	2.5 [1.0–4.0]
Diameter (mm)	7 [4.5–10.0]

Abbreviations: T: tumor, R0: complete resection, R1: microscopic resection, R2: macroscopic resection, primary acquired melanosis (PAM)

### Outcomes

Mean follow up was 4.7 years (interquartile range –IQR- from 1.3 to 6.9; median 2.7 years), with 34 patients followed for less than 2 years. At maximal follow-up, crude local relapse rate was 27.8% (*n* = 25) with a median time to local relapse equal to 2.9 years (IQR: 1.5 to 4.7). The cumulative incidence of local failure at 2, 5 and 10 years was 10.6% [4.9%;18.8%], 33.2% [20.8%;46.1%] and 49.5% [32.3%;64.5%]. The patterns of local relapse were in field in 16.0% (*n* = 4), and marginal or out of field 52.0% (*n* = 14) but could not be assessed in 28.0% of the patients (*n* = 7). We could not identify a specific pattern of failure by bulbar site (versus non-bulbar) (*p* = 0.88) and epithelioid type (*p* = 0.29). Most out-of-field failures occurred in unirradiated ocular quadrants, in the conjunctival folds or at the corneal angle. Proton therapy reirradiation was performed in 14 patients (14/25 (56%) of local failures). Ultimate salvage treatment consisted of exenteration in 13 patients (13/25 (52%) of local failures) patients at relapse or various conservative treatments in the others.

Regional, distant failure and specific death rates were 4.4% (*n* = 4), 5.5% (*n* = 5) and 4.4% (n = 4), respectively. At maximal follow-up, 34 patients (37.0%) presented a failure and/or died corresponding to a progression-free survival of 84.2% [73.7%;90.7%] at 2 years and 61.5% [47.1%;73.1%] at 5 years. The cumulative incidence of cause-specific death at 2 and 5 years was 1.1% [0.1%;5.6%] and 3.6% [0.6%;11.9%].

#### Prognostic factors

**Fig. 1:**
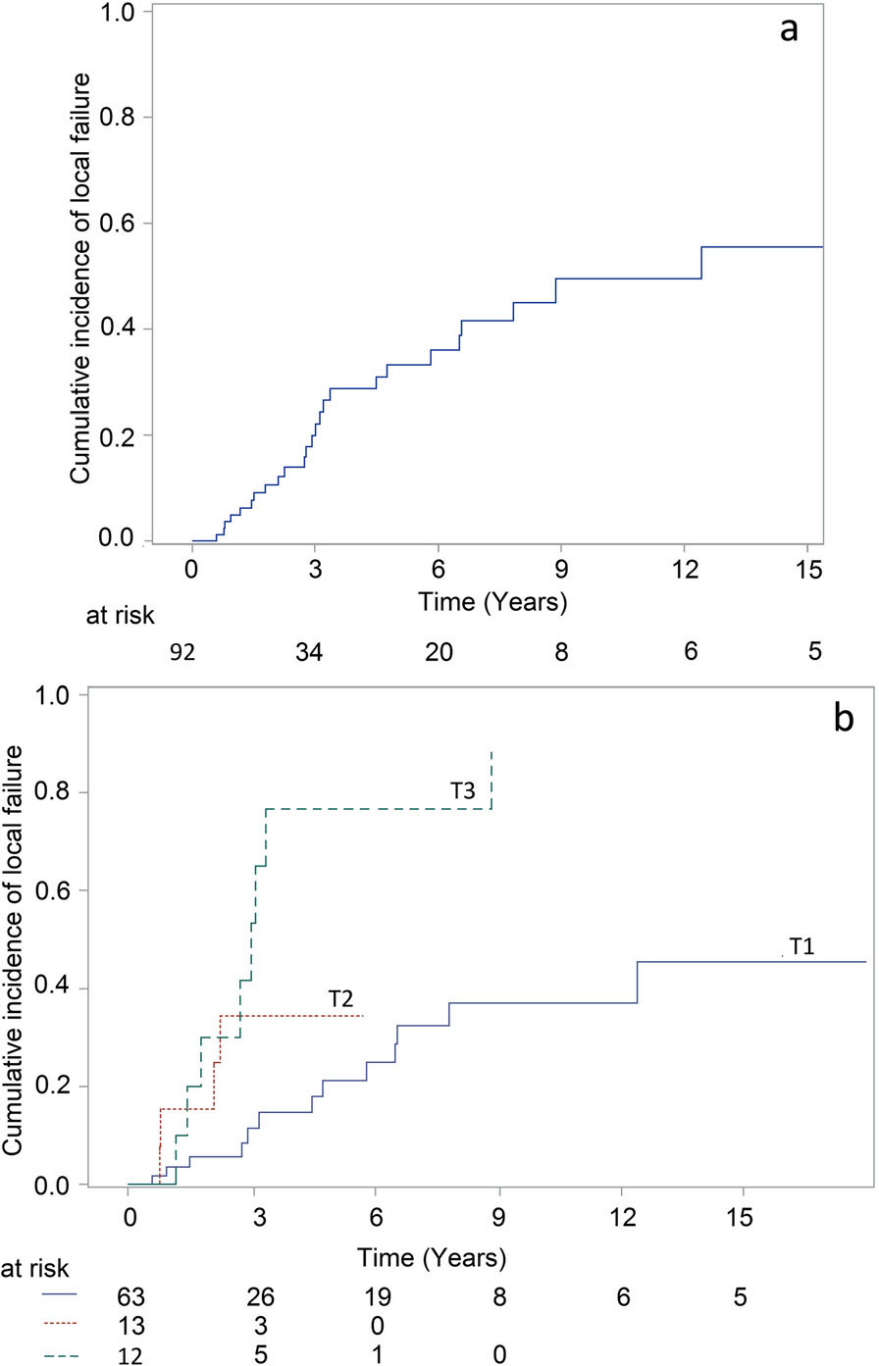
Cumulative incidence of local failure: (1a) for the 92 patients (1b) according to T stage

**Fig. 2:**
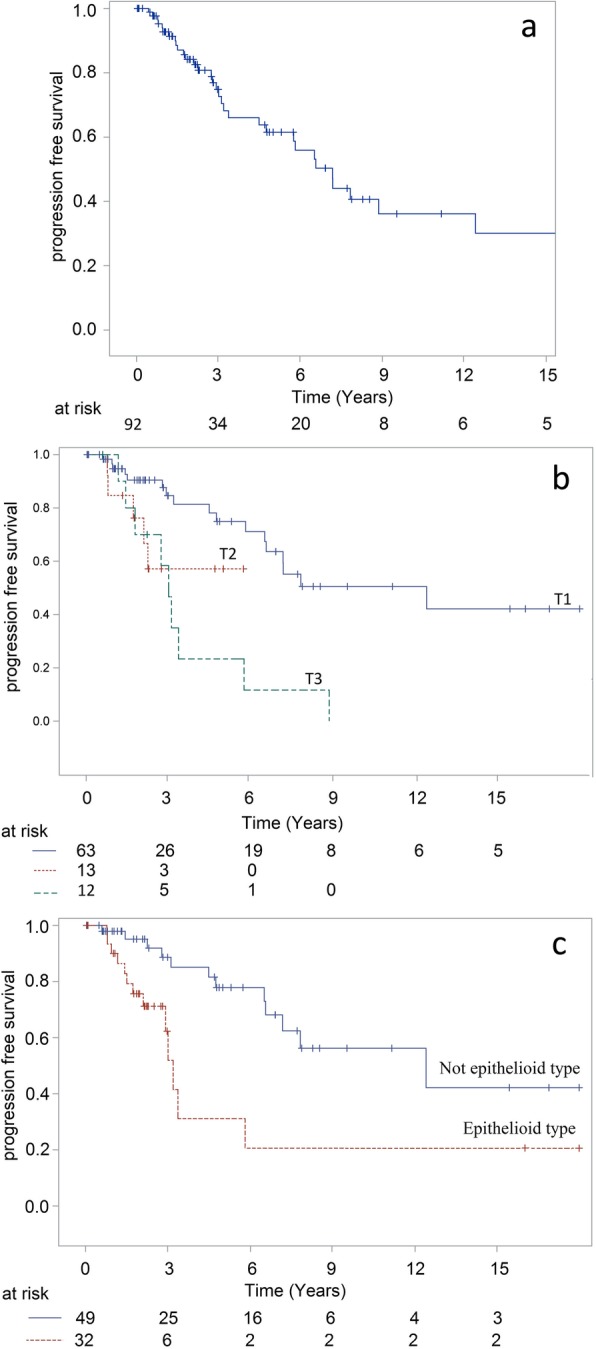
Progression free survival: (2a) for the 92 patients (2b) according to T stage (2c) according to epithelioid type

Tumor extent defined by clinical stage and quadrangular involvement were significant factor for local relapse (Table 2, Fig. 1: overall LC, + LC by T stage). Postoperative mitomycin (delivered in 90.9% of patients with PAM) was neither associated with better local control in the whole population (HR = 1.18[0.44;3.12), *p* = 0.74) nor in patients with PAM (HR = 1.03 [0.38;2.82], *p* = 0.94). First surgery at an outside institution was not significantly associated with poorer local control: cumulative incidence of local failure at 5 years was 24.3% [8.5%;44.5%] in case of surgery at our institution vs 38.7% [21.9%;55.2%] (HR = 1.41[0.62; 3.21], *p* = 0.41) in case of first surgery outside our institution. Clinical stage and epithelioid type were prognostic factors for poorer progression-free survival (Table 3, Fig. 2 by PFS overall and T stage + epithelioid type).

**Table 2:** prognostic factors of local relapse

	Bivariate analyses		Multivariate analyses	
	HR and 95% CI	*p*-value	HR and 95% CI	*p*-value
Female (vs Male)	1.39 [0,63; 3.05]	0.410		
Age	0.99 [0.96; 1.02]	0.556		
Precancerous lesion^a^				
PAM vs. de novo	1.81 [0.69; 4.74]	0.229		
Clinical stage				
T2 vs. T1	2.40 [0.74; 7.81]	0.146	2.56 [0.80; 5.83]	0.114
T3 vs. T1	4.37[1.84; 10.42]	0.001	7.32 [3.03; 17.67]	< 0.001
Pathological stage				
pT2 vs. pT1	2.69 [0.95; 7.64]	0.062		
pT3 vs. pT1	4.26[1.67; 10.88]	0.002		
Unifocal	0.56 [0.25; 1.28]	0.169		
Tumor epicenter At caruncle and/or conjunctival folds and/or lids vs. bulbar	4.85[1.62;14.53]	0.005		
Quadrangular involvement (degree)				
90 to 180 vs. < 90	1.97 [0.65; 5.96]	0.404	2.15 [0.79; 5.83]	0.133
> 180 vs. < 90	4.92 [1.43; 6.92]	0.028	4.00 [1.43; 11.16]	0.008
Epithelioid type	2.66 [1.12; 6.29]	0.026		
Margins				
R1 vs. R0	0.77 [0.35; 1.67]	0.507		
R2 vs. R0	1.23[0.14; 10.74]	0.854		
Number of mitoses> = 2	1.54 [0.72; 3.28]	0.265		
Ulceration	1.92 [0.65; 5.65]	0.234		
Thickness > = 2 mm	1.31 [0.47; 3.64]	0.605		
Diameter > = 7 mm	1.18 [0.55; 2.57]	0.667		
Treatment				
Mitomycin	1.41 [0.42; 4.78]	0.406		
Cryotherapy	0.68 [0.28; 1.68]	0.581		

^a^HR for nevus not computed since no events in this stratum

Abbreviations: T: tumor, R0: complete resection, R1: microscopic resection, R2: macroscopic resection, HR: hazard ratio; CI: Confidence interval; vs.: versus, primary acquired melanosis (PAM)

**Table 3:** prognostic factors of progression free survival

	Bivariate analyses		Multivariate analysis	
	HR and 95% CI	*p*-value	HR and 95% CI	*p*-value
Female (vs. Male)	1.42 [0,70; 2.87]	0.332		
Age	1.01 [0.98; 1.03]	0.574		
Pre- cancerous lesion				
nevus vs. de novo	0.18 [0.02; 1.47]	0.109		
PAM vs. de novo	1.40[0.57;3.42]	0.465		
Clinical stage				
T2 vs. T1	3.01 [1.04; 8.73]	0.042	3.19 [1.04; 9.80]	0.042
T3 vs. T1	4.33 [1.88; 9.97]	0.001	4.75 [1.47; 15.28]	0.009
Pathological stage				
pT2 vs. pT1	3.18 [1.16; 8.67]	0.024		
pT3 vs. pT1	4.32 [1.82; 10.22]	0.001		
Unifocal	0.87 [0.38;2.02]	0.751		
Tumor epicenter caruncle and conjunctival folds + lids vs. bulbar	2.95[1.24;7.03]	0.015		
Quadrangular involvement (degree)				
90 to 180 vs. < 90	1.26 [0.46; 3.47]	0.648		
> 180 vs. < 90	2.62 [0.75; 9.47]	0.132		
Epithelioid type	3.29 [1.44; 7.52]	0.005	2.68 [1.12; 6.43]	0.027
Margins				
R1 vs. R0	0.87 [0.42; 1.80]	0.704		
R2 vs. R0	0.89 [0.11; 7.03]	0.909		
Number of mitoses> = 2	1.09 [0.54; 2.18]	0.808		
Ulceration	3.10 [1.26; 7.62]	0.014		
Thickness > = 2 mm	1.06 [0.46; 2.46]	0.892		
Diameter > = 7 mm	1.14 [0.57; 2.29]	0.705		
Treatment				
Mitomycin	1.10 [0.35; 3.47]	0.875		
Cryotherapy	0.88 [0.37; 2.11]	0.775		

^a^*HR* for nevus not computed since no events in this stratum

Abbreviations: T: tumor, *R0*: complete resection, R1: microscopic resection, R2: macroscopic resection, HR: hazard ratio; CI: Confidence interval; vs.: versus, primary acquired melanosis (PAM)

#### Toxicity

During follow-up, cataract was reported in 22 patients (23.9%), and glaucoma in 13 patients (14.1%). Conjunctival, corneal thinning and scleral perforation were reported in 9, 11 and 1 patients (cumulated = 21 patients, 22.9%), respectively. Such trophic toxicity was higher in patients with cryotherapy (31.8%) compared to those undergoing postoperative mitomycin (0%) or neither cryotherapy nor mitomycin (1 patient, 7.7%), *p* < 0.01. The latter patient already had extraocular extension with scleral perforation at diagnosis. Conjunctival scarring was reported in seven patients (7.6%). Madarosis was reported in 21 patients (22.8%). Lacrymal duct stenosis and dry eye syndrome were reported in 5 (5.5%) and 28 patients (30.4%). Macular edema was noted in 1 patient and various other mild complications in 17 patients. The patient with a macular edema underwent topical anti-inflammatory agents. Severe trophic complications were managed with an amniotic membrane graft in 5 patients and a scleral graft in two. Surgery for hypertonia was performed in 2 patients. Exenteration was performed in 13 patients at relapse.

Visual acuity was assessed in 61 patients and remained stable or improved in 57.4% of patients. After adjusting on visual acuity at baseline, prognostic factors in multivariate linear regression for visual deterioration were age, tumor extent (multifocality, quadrangular involvement > 180 degrees) and cryotherapy (Table 4).

**Table 4:** Prognostic factors of visual acuity deterioration expressed in logMar from baseline value using linear regression on the 61 patients with available data on visual outcomes

	Bivariate analyses^a^		Multivariate analysis ^a ∆^	
	Estimation^◊^	*p*-value	Estimation^◊^	*p*-value
female (vs. male)	0.046(0.164)	0.781		
Age (years)	0.010(0.005)	0.072	0.01 (0.004)	0.008
Precancerous lesion nevus vs. de novo PAM vs. de novo	0.146(0.180) 0. 365 (0.184)	0.423 0.052	0.243 (0.168) 0.285 (0.126)	0.153 0.028
Clinical stage				
T2 vs. T1	0.040(0.214)	0.853		
T3 vs. T1	0.524 (0.333)	0.121		
Pathological stage				
pT2 vs. pT1	0.031(0.203)	0.880		
pT3 vs. pT1	0.723(0.375)	0.059		
Multifocal	0.768(0.202)	< 0.001	0.554 (0.157)	< 0.001
Quandrangular involvement from 90 to 180° vs < 90° > 180° vs < 90°	1.446(0.284) 0.246(0.146)	0.098 < .001	0.176 (0.115) 0.954 (0.237)	0.130 < 0.001
Epithelioid type	0.123(0.166)	0.461		
Margins				
R1 vs.R0	0.041(0.181)	0.8193		
R2 vs.R0	−0.302(0.251)	0.2329		
Number of mitoses < 2	−0.003(0.159)	0.986		
Ulceration	0.197(0.236)	0.407		
Thickness < 2 mm	0.147(0.248)	0.556		
Diameter < 7 mm	−.020(0.161)	0.899		
Cryotherapy	0.585(0.149)	< 0.001	0.461 (0.11)	< 0.001
Mitomycin	−0.302(0.170)	0.082		
Follow-up time (years)	0.007(0.013)	0.607		

^a^Adjusted on visual acuity at baseline

^∆^ associated R-square (percentage of explained variance) was 0.64

^◊^Estimation expressed as estimated beta value and standard deviation from linear regression. For a qualitative parameter, a beta value of 0.3 logMAR was considered as significant deterioration in visual acuity

Abbreviations: T: tumor, *R0*: complete resection, *R1*: microscopic resection, *R2*: macroscopic resection, HR: hazard ratio; *CI*: Confidence interval; vs.: versus, primary acquired melanosis (PAM)

## Discussion

Large series of conjunctival treated with no touch surgery and proton therapy are rare and visual outcomes are rarely documented in this situation. In this series of conjunctival melanomas, 92 patients were treated with conservative surgery and proton therapy targeting areas at risk for relapse on the ocular surface, conjunctival folds or tarsal conjunctiva. Two thirds of the patients also received cryotherapy perioperatively (to secure resection margins) or mitomycin postoperatively (to treat PAM, which is not included in the radiation fields). Eye preservation has been advocated since the 2000’s using an association of no touch surgery and cryotherapy of resection margins [8]. However, rates of relapses and related deaths [22], as well as local failure, remain substantial [23].

Attempts to reduce local relapse rates have used postoperative mitomycin and other topical chemotherapies [24]. Efficacy is however limited by the small penetration depth of these topical treatments and local relapse rates remain about 40–50% [24]. Radiation therapy (by brachytherapy or external beam modalities) also appears to decrease the risk of relapse [6, 10, 21, 25–30]. We here report 5-year and 10-year local failure incidences of 33.2 and 49.5% with proton therapy for conjunctival melanomas, which is consistent with the literature [2] [13, 27, 31]. Knowing the incidences of local failure, patients treated for conjunctival melanomas should have a tight follow-up schedule. Patients are asked to see their general ophthalmologist every 3 months for 2 years, every 6 months for 3 additional years, then annually. The patients are also asked to see their onco-ophthalmologist every 6 months during the first 2 years, and every year for 3 additional years.

Moreover, salvage conservative local treatment using proton therapy reirradiation was performed in 15% of the patients, i.e. in 56% of patients experiencing local failure. Thus, we were able to preserve eyes and vision in a half the patients with tumor relapse (similar to Shields’ [23]). It was so without compromising survival as deaths from conjunctival melanoma occurred in 4.4% of the patients.

Most local failures occurred out of the radiation field, and neither cryotherapy (of resection margins) nor mitomycin (covering the whole ocular surface, with PAM not included in radiation fields while invasive melanoma was) were protective factors for local failure. Out-of-field failures occurred in unirradiated ocular quadrants are consistent with the literature [26]. Enlarging radiation fields is however at risk of significantly increasing the probability of severe toxicity. Severe dry eye syndrome and limbal stem cell deficiency as a consequence of corneal vascularization are among these severe toxicities [21]. Thus, it may be worth reinforcing quality assurance of surgery to avoid tumor spread by strict compliance with the no touch technique, changes of instruments and not using conjunctival anesthesia but rather general anesthesia. Means to accurately define margins for proton therapy, such as dermoscopy [32] and impact of time between surgery and proton therapy might also be worth investigating. Finally, it might be preferred to limit the radiation fields to limit radiation-induced toxicities, with the idea that out-of-field failures may be retreated if necessary, as done in 56% of our patients.

In our series, most frequent toxicities were trophic toxicity by corneal/conjunctival/scleral thinning and madarosis, occurring in almost a fourth of the patients. Trophic toxicity was high in patients undergoing cryotherapy, which was abandoned after observation of these toxicities in the early years of the study. Despite cataracts in 23.9% and dry eye in 30.4% of the patients, 57% of them had stable or improved visual acuity despite treatment. Factors for visual deterioration were related to tumor extent. Finally, radiation therapy by either brachytherapy or proton therapy appears to not only reduce relapse rates but also to allow good visual acuity [26, 28, 29]. It is appropriate to treat extended forms of conjunctival melanomas either in depth (T2–3) or superficially (quadrants T1b-c) [21] and it can also treat ulcerative forms [30]. T3 tumors and complex tumor shapes including the conjunctival folds, caruncle or tarsal conjunctiva may not be easily treated with brachytherapy. In such cases, proton therapy is an appropriate technique [21]. As quality of initial surgery, i.e. a no touch surgery technique, is a major prognostic factor [22, 25, 33] [28], all patients underwent no touch surgery but in 47.2% of them, some form of surgery had been performed before referral and quality of first surgery could not be assessed [28]. There were 24% local relapses 5 years after first surgery at our institution and 39% when surgery was done before referral. Epithelioid type only was identified as a prognostic factor for progression-free survival. Distant metastasis rates and related-death rates were inferior in our series compared to others. While there are no clear explanations for this, it is noticeable that published series vary by the presence of precursor lesions. A majority of our patients had melanoma with PAM. These proportions are consistent with those of large series by Shields et al in which melanomas were associated with PAM (74%), from pre-existing nevus (7%), and de novo (19%) [23]. The presence of these conjunctival melanoma precursors is associated with different prognoses (de novo doing worse [23]. In our series, advanced T stage [16] was a more powerful poor prognosis factor. Other commonly found criteria, such as non-limbal location, were significant on bivariate analyses only [23, 34].

Visual outcomes were overall favorable and provide new data as we are not aware of large series of patients with melanomas and visual outcomes after proton therapy in other series.

This series has the drawbacks of retrospective monocentric series, but all patients underwent surgery and proton therapy*.* Like the no-touch technique, sampling and analyses of pathology specimen require expertise. In our experience, reports from outside institutions are very rarely informative with respect to thickness. Thickness may determine the need for sentinel node sampling, that was not performed systematically at our center. However, this issue remains debated and our regional failure rates were low despite long follow-up, suggesting that sentinel node sampling might not be systematically needed. Another limitation is the absence of BRAF staining [35, 36] and search for other biomarkers associated with prognosis [37] including immune markers [38]. Of note, we are currently conducting parallel multicentric immunostaining studies on operative samples. Furthermore, patients were variably treated with mutually exclusive cryotherapy or mitomycin depending on the period of treatment. However, these treatments had no effect on local failure or PFS in a series of patients homogenously treated with surgery and proton therapy.

## Conclusion

The cumulative incidence of local failure after proton therapy following surgery at 5 years was 33.2%. Proton therapy allowed an efficient conservative ultimate treatment at relapse in about half the relapses. Outcomes compare favorably with the literature with visual preservation in most cases and manageable toxicities, requiring surgical intervention in limited numbers of patients.

## Data Availability

yes, if requested.
